# Medical imaging-derived artificial intelligence for prognostic stratification and treatment response prediction in interventional therapy of hepatocellular carcinoma

**DOI:** 10.1016/j.iliver.2026.100240

**Published:** 2026-05-12

**Authors:** Ying Lei, Tianyi Xia, Yawen Wang, Xinyu Zhou, Xinyu Gao, Shenghong Ju

**Affiliations:** Nurturing Center of Jiangsu Province for the State Laboratory of AI Imaging and Interventional Radiology, Department of Radiology, Zhongda Hospital, School of Medicine, Southeast University, Nanjing 210009, Jiangsu, China

**Keywords:** Hepatocellular carcinoma, Artificial intelligence, Interventional therapy, Prognostic stratification, Treatment response prediction

## Abstract

Hepatocellular carcinoma (HCC) is a malignant tumor that is common worldwide. It is characterized by high incidence and mortality rates. Interventional therapy is a minimally invasive treatment for HCC that offers diverse methods that cover different stages. Because of the significant heterogeneity of tumors, even at the same stage, the effectiveness of interventional therapy can vary greatly, which makes it difficult for clinicians to determine the optimal treatment plan before treatment. Increasing evidence suggests that tumor-related imaging characteristics are correlated with biological functions and can be used to predict different subtypes of HCC and reflect their heterogeneity. In recent years, artificial intelligence (AI) has received widespread attention and been applied widely. AI can automatically extract features from medical images, objectively quantifying low-dimensional to high-dimensional information about tumors, which helps to directly or indirectly predict prognostic stratification and treatment response to interventional therapy. Furthermore, when AI integrates high-dimensional quantifiable information from imaging data with multimodal clinical and molecular data, its accuracy and interpretability improve significantly. Although image-derived AI models have achieved good performance and have broad prospects for application in the prognosis and treatment of HCC, their clinical implementation has limitations, including data and imaging standardization, model interpretability, and the need for multicenter validation. This review summarizes the latest advancements in medical image-driven AI in the prognostic stratification and efficacy prediction of interventional therapy for HCC, and outlines the main challenges that need to be addressed and good prospects for application.

## 1 Introduction

**Fig. 1:**
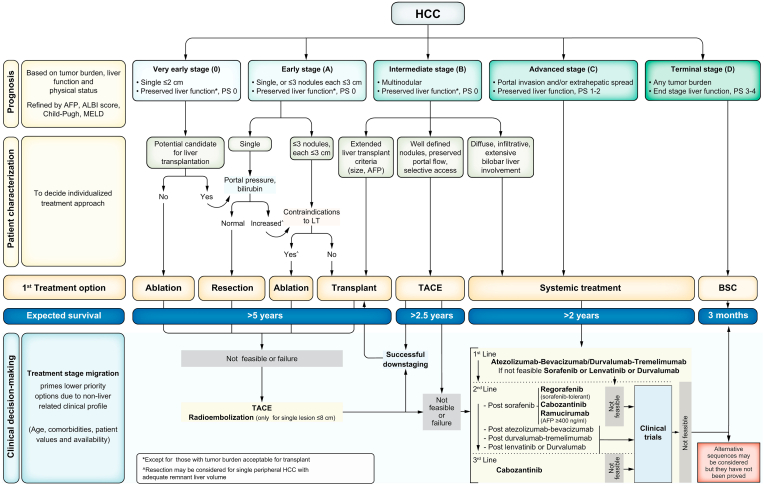
BCLC staging and treatment strategy in 2022 (Reprinted from Reig M et al.,^4^ with permission. License Number: 6178710056427). The BCLC system establishes a prognosis in accordance with the five stages that are linked to first-line treatment recommendations. The expected outcome is expressed as the median survival of each tumor stage according to available scientific evidence. Individualized clinical decision-making, according to data available on September 15, 2021, is defined by teams responsible for integrating all available data with the individual patient’s medical profile. Note that liver function should be evaluated beyond the conventional Child–Pugh staging. Full availability of data from the trial testing of the combination of tremelimumab and durvalumab may lead to these agents being incorporated as a first-line alternative. Abbreviations: AFP, alpha-fetoprotein; ALBI, albumin-bilirubin; BCLC, Barcelona Clinic Liver Cancer; BSC, best supportive care; ECOG PS, Eastern Cooperative Oncology Group-performance status; LT, liver transplantation; MELD, model of end-stage liver disease; TACE, transarterial chemoembolization.

Primary liver cancer ranks sixth in incidence and third in mortality globally, with approximately 865,000 cases and 758,000 deaths in 2022.^1^ Hepatocellular carcinoma (HCC) is the predominant subtype, accounting for 75% of cases.^2^ Survival outcomes have improved through advanced staging and treatments.^3^ The Barcelona Clinic Liver Cancer (BCLC) system remains the standard for categorizing HCC into five stages (Fig. 1).^4^ While surgical resection and transplantation are curative,^5^ less than 20% of patients qualify due to tumor burden or poor liver reserve, leaving 80% to rely on interventional therapy.^6^

Technological advancements in microwave ablation (MWA), transarterial radioembolization (TARE), and chemoembolization (TACE) have continuously improved prognosis.^7^ MWA is preferred for its efficiency and reduced “heat sink effect”.^6^^,^^8^^,^^9^ TARE has demonstrated superior disease control and survival benefits, particularly in transplant candidates, as seen in the LEGACY trial.^10^, ^11^, ^12^, ^13^ TACE remains a pivotal palliative intervention and the standard for unresectable intermediate-stage HCC.^14^, ^15^, ^16^ While drug-eluting bead (DEB)-TACE offers certain advantages, its superiority over conventional TACE (cTACE) remains debated.^17^, ^18^, ^19^ Recent trials emphasize the need for individualized decisions beyond simple staging.^20^

**Fig. 2:**
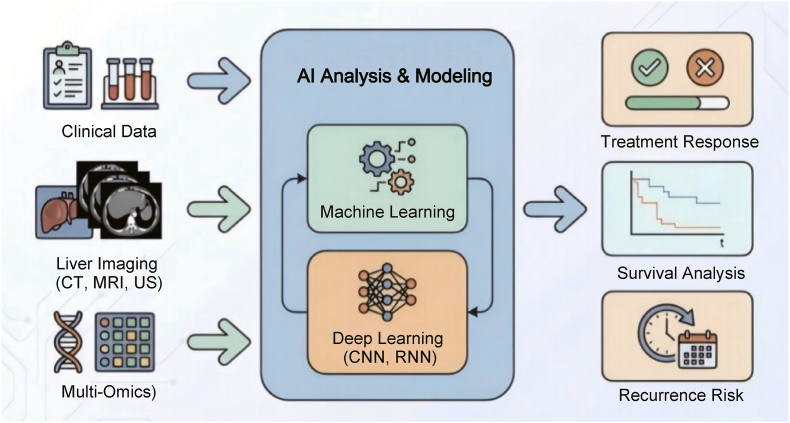
Al-driven prediction of outcomes in HCC interventional therapy. Generated by Gemini (Nano Banana 2 model, Google), based on user-defined prompts, and manually refined for clinical accuracy. Abbreviations: CT, computed tomography; MRI, magnetic resonance imaging; US, ultrasound; AI, artificial intelligence; CNN, convolutional neural network; RNN, recurrent neural network.

Interventional therapy supports various disease stages, yet no specific system currently exists to predict treatment outcomes.^21^, ^22^, ^23^, ^24^ Imaging remains central to HCC management, with Liver Imaging Reporting and Data System (LI-RADS) providing a standardized framework for diagnosis and evaluation.^25^, ^26^, ^27^, ^28^ However, traditional semantic features rely on manual interpretation, which is prone to observer variability.^26^ Furthermore, the complexity of preoperative clinical and molecular data can challenge even experienced clinicians. Artificial intelligence (AI), specifically machine learning (ML), optimizes performance through data-driven modeling.^29^ Deep learning (DL) autonomously extracts subvisual imaging phenotypes beyond predefined labels, minimizing prediction errors.^29^^,^^30^ By integrating imaging, clinical, and genomic data, AI models facilitate robust risk-prediction frameworks for individualized surveillance and treatment selection.^31^ AI workflows are now being integrated into all stages of HCC management (Fig. 2).^31^

With the continuous advancement and widespread application of interventional therapy in cancer treatment, the accurate identification of patients who are suitable for and can benefit from this therapy using AI has become a major challenge. Currently, AI is being applied to the interventional treatment of HCC in multiple studies, which is expected to enhance treatment effectiveness in the future. In this review, we summarize existing AI models that are directly or indirectly used to predict the response and prognosis of HCC interventional therapy driven by imaging. Furthermore, several research directions are proposed for addressing the challenges faced by such models, particularly issues such as standardization and lack of interpretability, to aid their rapid adaptation to clinical practice.

## 2 Bibliometric analysis of AI in the imaging of HCC treated by interventional therapy

AI can extract and process high-dimensional features from imaging data before and after interventional therapy, ultimately generating personalized prediction results that clinicians can refer to. To enable a comprehensive understanding of the current research status in this field, the Web of Science Core Collection database was searched on October 1, 2025. The search strategy focused on three major themes using keywords including, but not limited to: (1) HCC and related terms like “liver cancer”; (2) AI and its subfields such as DL and radiomics; and (3) interventional therapies including transarterial chemoembolization (TACE) and ablation techniques. After the aforementioned themes were combined using the “AND” operator, the search scope was limited to English-language literature published between January 1, 2000, and January 1, 2025. A total of 267 articles were retrieved initially. After detailed screening, 226 directly relevant original research articles were included. A bibliometric analysis centered on the review topic was conducted using VOSviewer (version 1.6.20)^32^ and CiteSpace (version 6.3.1).^33^^,^^34^

**Fig. 3:**
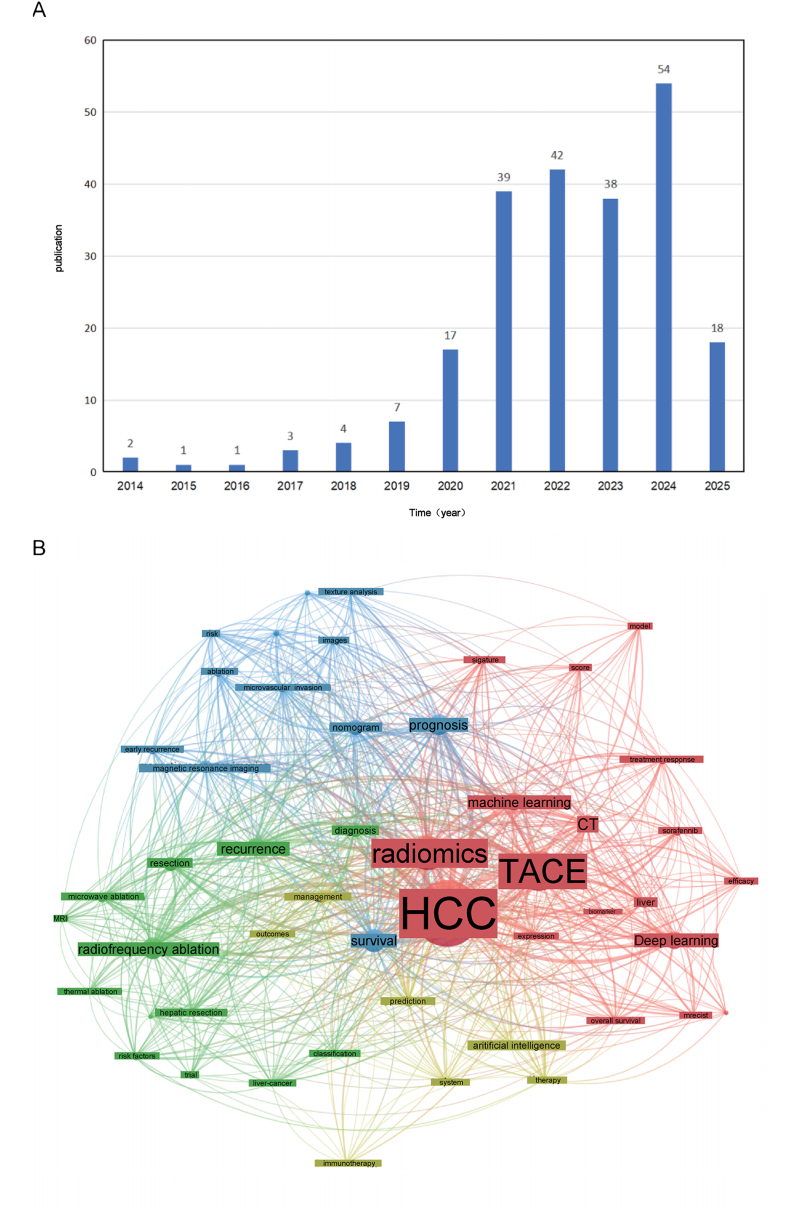
Bibliometric analysis of the literature on interventional therapy for HCC based on DL and radiomics methods. (A) Trend chart of the number of published papers. (B) Co-occurrence network diagram of relevant keywords. Abbreviations: HCC, hepatocellular carcinoma; CT, computed tomography; MRI, Magnetic Resonance Imaging; TACE, transarterial chemoembolization.

Relevant literature was included, involving 1729 authors and 482 research institutions distributed across 28 countries. The research findings were published in 109 academic journals with 5635 cited references. Research on AI in interventional therapy for HCC began in 2014. Overall, the number of publications in this field showed a continuous upward trend, particularly after 2019, when a rapid increase in publication volume occurred, which indicates that this field became an emerging research area of great interest (Fig. 3).

In terms of output activity, the *Journal of Hepatocellular Carcinoma* ranks first with 27 publications, followed by *Frontiers in Oncology* with 15 publications and *Abdominal Radiology* with 10 publications. In terms of academic influence, the pattern was different. Although *European Radiology* only published seven articles, it received 505 citations, with its average influence per article far ahead, ranking first. The included original research articles cited a large number of high-quality journal articles, with the most cited being the *Journal of Hepatology* with 431 citations, followed by *Radiology* (346 citations) and *Hepatology* (333 citations). Overall, the application of radiomics and DL in HCC has become a popular research direction in the fields of hepatology, oncology, and radiology. This pattern indicates that this field is moving in an interdisciplinary direction that deeply integrates the clinical theory of hepatology with interventional techniques of radiology, and its development is highly dependent on the progress of basic disciplines, achieving knowledge innovation and integration in specialized journals.

Co-occurrence network analysis was conducted based on the top 50 most frequently occurring keywords, identifying several major clusters, including “HCC”, “transarterial chemoembolization”, “radiomics,” “survival analysis” and “prognostic assessment” (Fig. 3). Among them, “HCC” occupies a central position, with an appearance frequency of 186 and total link strength of 788; “TACE” as a key treatment method, appears 96 times, with a link strength of 423; and “radiomics” emerges as an important branch in methodology, with an appearance frequency of 79 and total link strength of 394. “Deep learning” (33 times, link strength 137) and “machine learning” (33 times, link strength 135), as two parallel paradigms of AI technology, jointly drive methodological innovation. Notably, “nomogram” appears 29 times as a clinical predictive tool, with a link strength of 160, which reflects the trend of research outcomes translating into clinical practice. In terms of clinical issues and study endpoints, “survival” “recurrence” and “prognosis” constitute the core concerns. Furthermore, “microvascular invasion” as a key pathological feature affecting prognosis, despite its relatively low appearance frequency (14 times), still has a link strength of 93. Its preoperative non-invasive prediction is an important challenge in this field. To summarize, AI in the field of liver cancer interventional therapy has formed a highly cohesive research paradigm that focuses on survival, prognosis, and microvascular invasion (MVI) prediction, with “HCC” as the research object, “TACE” as the main treatment background, and “radiomics” and “deep learning” as the core methods. This reflects the strong clinical orientation and interdisciplinary integration of this field, providing important references for future research.

## 3 AI in predictive tasks and applications for interventional treatment of HCC

Building AI prediction models involves data acquisition, feature selection, and model construction, among other steps. These models typically use pre-treatment clinical data (demographics and labs) and imaging (via radiomics and DL) to predict endpoints such as response, survival, or complications. Given the heterogeneity of HCC and diversity of interventional therapies, accurate pre-treatment prediction is crucial for individualized strategy selection. It covers the application of AI in major interventional therapy methods, including the role and comparison of AI in TACE, ablation, TARE, hepatic artery infusion chemotherapy (HAIC), and combination therapies. TACE and local ablation are the most common interventions. Recent studies are summarized in Table 1, Table 2.

**Table 1:** Most recent studies that use AI to predict outcomes of TACE for HCC.

Author, Year	Treatment	No.of participants	Outcomes predicted	Input	Methods		Best model performance	
					Feature selection	Model construction	Training	Test
Wei et al., 2025^35^	TACE	2333	ASPFS	Clinical data Semantic features	cML	CatBoost	AUC: 0.970	AUC: 0.94/0.93
Hao et al., 2025^36^	TACE	2068	ER	Clinical data Semantic features	cML	XGBoost	AUC: 0.904	AUC: 0.888/0.854
Zhang et al., 2024^37^	DEB-TACE	108	TR (mRECIST)	Clinical data Semantic features CE-CT (radiomics)	cML	LR	AUC: 0.860	AUC: 0.927
Zhang et al., 2024^38^	cTACE	367	TR (mRECIST)	Clinical data Semantic features CE-CT (radiomics)	cML	RF	AUC: 0.826	AUC: 0.800
Chen et al., 2024^39^	TACE	1695	OS	Clinical data Semantic features	—	DeepSurv neural network	C-index: 0.74	C-index: 0.69/0.70
Wang et al., 2023^40^	TACE	243	TACE unsuitability	Clinical data Semantic features	cML	XGBoost	AUC: 0.906	AUC: 0.894
Li et al., 2023^41^	DEB-TACE	288	ALFD	Clinical data Semantic features	cML	nomogram	AUC: 0.762	AUC: 0.878
Wang et al., 2022^42^	TACE	543	TTP	Clinical data Semantic features CE-CT (radiomics)	cML	EfficientNetV2	1-year AUC: 0.822	1-year AUC: 0.720
Ren et al., 2022^43^	TACE with TKI	103	TR (mRECIST)	Clinical data Semantic features CE-CT (radiomics)	cML	RMNN	AUC: 0.940	—
Li et al., 2022^44^	TACE	248	OS	Clinical data Semantic features CE-CT (DL)	DL	Multi-DL	—	AUC: 0.871 Accuracy: 83.9%
Niu et al., 2021^45^	TACE	461	TACE refractoriness	Clinical data Semantic features CE-CT (DL)	cML	nomogram	C-index: 0.844	C-index: 0.831
Kong et al., 2021^46^	TACE	99	TR (mRECIST)	Clinical data Semantic features MR (radiomics)	cML	nomogram	AUC: 0.861	AUC: 0.884
Peng et al., 2020^47^	TACE	562	TR (mRECIST)	CE-CT (DL)	DL	ResNet50	AUC: 0.950–0.970	AUC: 0.94-0.98
Liu et al., 2020^48^	TACE	130	TR (mRECIST)	CE-US(DL)	DL	3D CNN	AUC: 0.980	AUC: 0.93
Morshid et al., 2019^49^	TACE	105	TACE refractoriness	Clinical data Semantic features CE-CT (DL)	cML	RF	AUC: 0.730 Accuracy: 74.2%	—

Abbreviations: TACE, transarterial chemoembolization; cTACE, conventional TACE; DEB-TACE, drug eluting beads TACE; TKI, tyrosine kinase inhibitor; ASPFS, advanced-stage progression-free survival; ER, early recurrence; TR, treatment response; mRECIST, modified Response Evaluation Criteria in Solid Tumors; RFS, recurrence free survival; OS, overall survival; TTP, time-to-progression; ALFD, acute liver function deterioration; MRI, magnetic resonance imaging; CE-CT, contrast-enhanced computed tomography; CE-US, contrast-enhanced ultrasound; cML, conventional machine learning; DL, deep learning, AUC, area under the curve; CatBoost, categorical gradient boosting; XGBoost, extreme gradient boosting; LR, logistic regression; RF, random forest; RMNN, Resnet50_MIM_Nearest Neighbors; CNN, convolutional neural network.

**Table 2:** Most recent studies that use AI to predict outcomes of ablation for HCC.

Author, Year	Treatment	No.of participants	Outcomes predicted	Input	Methods		Best model performance	
					Feature selection	Model construction	Training	Test
Li et al., 2025^50^	Ablation	288	ER	Clinical data CE-CT (radiomics/DL)	cML	RF + LR + KNN + LGBM	AUC: 0.981	AUC: 0.910/0.851
Li et al., 2025^51^	Ablation	288	ER	CE-CT (radiomics/DL)	cML	LGBM	AUC: 0.924	AUC: 0.899/0.839
Kong et al., 2025^52^	TA	289	ER	Clinical data MRI (radiomics)	cML	CNN/RNN + Extra Trees	AUC: 0.931	AUC: 0.740
Zhang et al., 2024^53^	Ablation	751	OS	Clinical data Semantic features	Standard statistical method	Aorsf	C/D AUC: 0.733 C-index:0.736	C/D AUC: 0.733 C-index: 0.793
Zhang et al., 2024^54^	Ablation	898	RFS	Clinical data Semantic features	Standard statistical method	nomogram	C-index: 0.694	C-index: 0.651
Wang et al., 2024^55^	TA	535	ER	Clinical data Semantic features MRI(radiomics/DL)	cML	LR	AUC: 0.794	AUC: 0.777/0.787
Hamed et al., 2024^56^	RFA	111	A favorable outcome	Clinical data Semantic features	cML	XGBoost	AUC: 0.950	AUC: 0.80
Wu et al., 2022^57^	MWA	513	RFS	Clinical data Semantic features CE-US(radiomics/DL)	cML DL	ResNet 18+ ML	C-index: 0.633	C-index: 0.721
Sato et al., 2022^58^	RFA	1778	recurrence	Clinical data Semantic features	cML	GBDT	C-index: 0.720	C-index: 0.67
An et al., 2022^59^	MWA	1574	ER	Clinical data Semantic features	Standard statistical method	XGBoost	AUC: 0.750	AUC: 0.74/076
Lv et al., 2021^60^	RFA	891	AIR	Clinical data Semantic features MR(radiomics)	cML	nomogram	AUC: 0.941	AUC: 0.818

Abbreviations: RFA, radiofrequency ablation; MWA, microwave ablation; TA, thermal ablation; HCC, hepatocellular carcinoma; RFS, recurrence free survival; ER, early recurrence; OS, overall survival; AIR, aggressive intrasegmental recurrence; CE-MRI, contrast-enhanced magnetic resonance imaging; CE-CT, contrast-enhanced computed tomography; CE-US, contrast-enhanced ultrasound; cML, conventional machine learning; DL, deep learning; AUC, area under the curve; C/D AUC, concordance-discordance AUC; C-index, concordance index; LR, logistic regression; RF, random forest; KNN, *K*-nearest neighbor; LGBM, light gradient boosting machine; RNN, recurrent neural network; CNN, convolutional neural network; GBDT, gradient boosting decision tree; XGBoost, extreme gradient boosting.

### 3.1 AI in the prediction of the efficacy of TACE

Several meta-analyses have assessed AI models for TACE outcomes. Soni et al. reviewed machine learning models in intermediate-stage HCC and found better prediction for objective response (area under the curve [AUC] 0.85) than 3-year overall survival (OS) (AUC 0.70).^61^ Kiani et al. compared DL and handcrafted radiomics models across 27 studies. The researchers noted no significant performance difference and emphasized task-specific feature selection.^62^ Keshavarz et al. showed that integrating clinical and radiological features consistently improves prediction.^63^

Regarding specific subgroups, researchers have shown that HCC patients with complications such as diabetes mellitus or portal vein tumor thrombus (PVTT) have poorer survival after TACE.^64^, ^65^, ^66^ Several research groups have focused on these high-risk groups. Wu et al. retrospectively analyzed 636 HCC patients with type 2 diabetes mellitus from four medical centers and constructed five machine learning models. The random survival forest (RSF) achieved the best performance, with AUCs of 0.862, 0.815, and 0.798 for 1-, 2-, and 3-year survival in the external validation cohort, respectively.^67^ Lu et al. targeted PVTT patients undergoing TACE, lenvatinib, and programmed cell death protein 1 (PD-1) inhibitor (TLP) therapy, creating a combined clinical-radiomics model that achieved AUCs of 0.95 and 0.84 for early treatment response prediction.^68^ Xiong et al. developed and validated a nomogram to predict post-treatment recurrence risk in Hepatitis B virus (HBV)-related HCC with high hepatitis B surface antigen (HBsAg) levels (≥ 1000 U/L), and obtained C-indices of 0.682, 0.666, and 0.740 for the training, internal, and external validation cohorts, respectively.^69^

From a methodological perspective, DL has emerged as a key driver for the precision prediction of TACE outcomes, although challenges such as data diversity and model interpretability remain. Predictions using digital subtraction angiography (DSA) data are rare. Zhang et al. proposed DSA-Net using DSA videos for real-time response prediction, with Dice scores of 0.75/0.73.^70^ To balance interpretability and generalizability, some researchers have combined radiomics with DL. Cen et al. established a prognostic model by simultaneously integrating radiomics and DL.^71^ After comparing the predictive performance of various models, the researchers found that the combined model, including five radiological features of arterial phases, DL score, and HBsAg, exhibited the best predictive ability. Notably, some researchers have attempted to combine DL with radiomics to form so-called “DL radiomics”. This method uses convolutional neural networks (CNNs) to extract radiomics features at the DL level, followed by traditional radiomics processes for feature selection and model construction. Yin et al. developed and validated a DL radiomics model based on CT for predicting treatment response and PFS in patients with unresectable HCC treated with a combination of TACE, HAIC, PD-1 inhibitors, and tyrosine kinase inhibitors (TKIs).^72^ The study was based on a pretrained ResNet50 using two input channels (images and clinical features) to predict the efficacy of tumor treatment according to the modified Response Evaluation Criteria in Solid Tumors (mRECIST) evaluation criteria. Li et al. developed and validated an automatic segmentation model based on DL, combined with radiomics, to predict post-TACE liver failure (PTLF) in patients with HCC.^73^ Lin et al. used a DL model (ResNet50) to extract features from contrast-enhanced CT images before TACE treatment and constructed a machine learning model to predict TACE responses.^74^ The AUC values of the external testing dataset reached 0.89–0.91 across multiple machine learning models.

Relying solely on single-time-point images may overlook treatment-induced dynamic changes; thus, longitudinal and multi-time-point modeling have gained attention. Wei et al. were the first to combine longitudinal contrast-enhanced magnetic resonance imaging (CE-MRI) data with multi-modal DL and machine learning techniques, using a dual two-dimensional ResNet50 structure and a transformer for feature integration to develop a long-term tumor response model for predicting patients undergoing drug-eluting bead transarterial chemoembolization (DEB-TACE). The AUC reached 0.941 and 0.925 in the training and validation cohorts, respectively.^75^ Yao et al. applied the Swin Transformer to multi-time-point MRI for TACE ± MWA and stratified patients into four risk groups (accuracy 0.87, AUC 0.92).^76^

Multi-omics is expected to deepen the understanding of HCC subtypes and optimize patient stratification, thereby facilitating individualized treatment. Radiogenomics integrates transcriptome, radiomics, and tumor immune microenvironment data to construct a “digital biopsy”. A common approach is to first establish and validate a prognostic model using AI and then compare model features with multi-omics data to enhance the biological interpretability of radiomics/DL features. Another type of research directly integrates multidimensional information, such as clinical pathology, gene mutations, DNA methylation, and immune composition, to identify and characterize HCC molecular subtypes. Subsequently, noninvasive imaging features are used as surrogate indicators of molecular and immune status to provide noninvasive support for precise classification and treatment. Wang et al. used preoperative CT images of HCC patients undergoing TACE treatment to determine radiological prognostic features. Simultaneously, based on the hypothesis that radiomics can reflect intratumoral heterogeneity, matched imaging and gene data were combined to establish a link between radiomics and gene expression profiles, and elucidate the biological basis of the association between individual radiomics phenotypes and survival outcomes in HCC.^77^ Additionally, some researchers have integrated multi-omics data such as genomics and transcriptomics, and identified two HCC subtypes (CS1 and CS2) with distinct clinical and biological characteristics using the MOVICS framework.^78^ Based on this, researchers integrated multiple machine learning models through the Mime framework, selected the optimal model, and developed an AI-derived risk score (AIDRS) that is easily translatable to clinical practice. This score significantly increased in patients with the more aggressive CS2 subtype, thereby demonstrating high consistency in risk stratification. Further functional experiments confirmed that the CEP55 gene is one of the core molecules that drives high AI-derived risk scores and CS2 subtype characteristics. Its knockdown significantly inhibited tumor proliferation, migration, and invasion, which validates the biological basis of this scoring system. Additionally, some researchers have also explored the transferability and validation of radiomics across different cohorts. For example, a CT-based proliferative HCC prediction model effectively stratified PFS in the TACE cohort.^79^ Similar features include the prediction of MVI and vessels enclosing tumor clusters, which are used to construct non-invasive imaging markers to prospectively predict patients’ potential responses to various treatment modalities such as TACE. 80, 81, 82 Overall, from systematic review evidence to specific populations, from single-time point to longitudinal, and from imaging to multi-omics, the clinical adaptability and interpretability of TACE prediction models are steadily improving.

To summarize, TACE prediction models range from “simple” radiomics to complex longitudinal/multi-omics approaches. Simple radiomics models (often combined with clinical data) are relatively mature, interpretable, and closer to clinical deployment for specific tasks. Their main limitation is generalizability across institutions. Complex models (DL on longitudinal imaging or multi-omics integration) are less mature, but promise to capture tumor heterogeneity and dynamic evolution, thereby enabling truly personalized therapy. The field is moving toward hybrid strategies that leverage both interpretability and predictive power, supported by rigorous multi-center validation.

### 3.2 Application of AI to the prediction of the efficacy of local ablation

Local ablation is an important radical treatment for BCLC stage 0-A HCC; however, the high recurrence rate makes risk stratification and follow-up optimization particularly crucial. Therefore, AI models tailored to survival and recurrence risks are continuously emerging. Shi et al. developed and validated a prediction model for OS after ablation therapy for HCC in male patients with chronic HBV infection who also have long-term smoking and drinking habits, with C-indices of 0.712 and 0.719 in the training and validation cohorts, respectively.^83^ Qiao et al. identified and evaluated various factors affecting the survival of elderly patients with cirrhotic HCC after ablation therapy, and constructed a nomogram to predict their 3-, 5-, and 8-year OS.^84^ Lu et al. conducted an in-depth analysis of the key factors affecting recurrence in HCC patients with high preoperative systemic immune-inflammation index levels after ablation therapy, and based on this, constructed a nomogram model to predict the RFS of patients.^85^ Other researchers identified gamma-glutamyl transferase, activated partial thromboplastin time (APTT), age, and alanine aminotransferase (ALT) as independent risk factors affecting recurrence in malnourished patients with HCC.^86^ The prognosis for patients with small HCC (≤ 3 cm) remains poor. Wen et al. constructed a radiomics nomogram based on preoperative MRI and platelet count to predict early recurrence (ER), and achieved an AUC of 0.981.^87^ However, there is currently no consensus on a monitoring strategy for initial recurrent HCC (irHCC). Conventional follow-up schemes with fixed intervals may fail to detect recurrence at an optimal time, thereby leading to delayed diagnosis. Chen et al. proposed and validated a new monitoring strategy for irHCC after ablation based on machine learning and dynamic risk prediction. The risk of tumor recurrence is calculated monthly using the RSF methodology and follow-up schedules are arranged to maximize the capability of relapse detection at each visit.^88^

Similar to research on TACE, that on ablation has gradually shifted from “preoperative static characteristics” to “postoperative dynamic integration”. Lim et al. used a deep CNN to focus on arterial enhancement lesions near the ablation zone after treatment, and distinguished them from local tumor progression (LTP) and other potentially enhancing lesions (including normal vessels or arterial port shunts) (AUC of 0.992), thereby reducing false positives and enhancing the saliency of the region of interest.^89^ An et al. evaluated the ablative margin (AM) after MWA for HCC based on a DL-based deformable image registration technique and analyzed the relationship between the AM and LTP, confirming that AM ≤ 5 mm and age > 65 years were independent risk factors for LTP.^90^ Shen et al. used changes in radiomics features from pre- and post-treatment CT images to predict ER after resection or ablation.^91^ In the validation set, the AUC of the radiomics algorithm (0.89) was significantly higher than that of the AFP change values (0.63). Luo et al. extracted delta radiological features from multiphase pre-ablation MRI, calculated the delta radiological score, and integrated this score with key clinical variables in an RSF model to predict RFS and OS.^92^ Huang et al. constructed a delta-radiomics nomogram based on multiphase CE-MRI, covering the tumor and a 10-mm area around it, to predict ER after percutaneous thermal ablation.^93^ Overall, the accuracy and applicability of ablation efficacy prediction are both improving for “intelligent post-operative image interpretation” and “multimodal fusion of delta features”.

### 3.3 Application of AI to the prediction of the efficacy of internal radiation therapy

In recent years, TARE has attracted widespread attention in the medical community as an innovative internal radiation therapy method. In this field, ^90^Y microspheres, an important therapeutic tool, have gradually demonstrated their unique advantages in the treatment of HCC. Sarioglu et al. constructed and evaluated a machine learning model based on pretreatment MRI radiomics features to predict the treatment response of large HCC (> 5 cm) to TARE.^94^ The researchers found that the radiomics model based on enhanced T1 images achieved the best predictive performance, with an accuracy rate close to 80%, AUC of up to 0.92, and specificity of 100%. Stocker et al. used pre-treatment MRI (emphasizing the portal venous phase) radiomics features combined with clinical parameters to predict the treatment response of HCC to radiation segmentectomy, a precise TARE technique.^95^ In the study, radiomics outperformed pure clinical models, with the best combination achieving an AUC of 0.736. Mansouri et al. were the first to systematically apply three quantitative analysis methods—“radiomics”, “dosimetrics” and dose-volume constraints—to predict the early response of HCC to ^90^Y selective internal radiation therapy. They innovatively used pretreatment simulation (99mTc-MAA) and post-treatment (^90^Y) SPECT/CT images to simultaneously extract features from both tumor and normal liver tissues, and constructed up to 1440 machine learning models. The “dosimetrics” model demonstrated remarkable predictive potential (AUC up to 1.0) and the features of normal liver tissues were found to be equally as important as those of the tumor.^96^ Mahmoud et al. extracted radiomics features from post-treatment MRI-T2 images to distinguish between HCC patients who responded and did not respond to ^90^Y TARE treatment. The model exhibited moderate predictive ability, but its overall performance was limited (AUC = 0.71).^97^ Marinelli et al. constructed a machine learning model that integrated radiomics features from baseline (pre-treatment) and early (1–2 months post-treatment) MRI to predict complete response after treatment.^98^ The model demonstrated excellent predictive performance in the independent validation set (AUC = 0.89), significantly outperforming models based on clinical characteristics and conventional imaging criteria (mRECIST). Aujay et al. developed a model to predict whether HCC achieves complete remission 4–6 months after ^90^Y TARE therapy. This model addresses the clinical pain point of difficulty in evaluating treatment efficacy caused by early inflammatory interference.^99^ A unique approach is body composition radiomics; sarcopenia and fat distribution (particularly visceral fat) are important factors affecting the prognosis of patients with HCC and are typically assessed through simple indicators such as cross-sectional area or average density. Saalfeld et al. applied radiomics to skeletal musculature and adipose tissues rather than the tumor itself, focusing on sarcopenia and fat distribution to predict the 1-year survival. This approach achieved a higher predictive value in patients treated with SIRT combined with sorafenib (AUC 0.80 vs. 0.76).^100^ Thus, it can be observed that the prediction of internal radiation therapy is advancing synergistically along multiple dimensions.

### 3.4 Application of AI in the prediction of the efficacy of combination therapy

In the context of the increasing popularity of combination and sequential treatments, AI is being used to identify “true beneficiaries”. Ding et al. constructed a CT-based clinical-radiomics nomogram to estimate the OS of patients with advanced HCC who received TACE in combination with camrelizumab and apatinib, with a Concordance index (C-index) of 0.840 and 1/2-year AUC of 0.936/0.946.^101^ Fang et al. constructed and validated a nomogram model combining MRI radiomics labels and clinical factors. This model can accurately and individually predict the PFS of patients with intermediate and advanced HCC who undergo TACE + RFA combination therapy.^102^ Researchers have confirmed that the interval between TACE and RFA (≤ 14 days) is an independent protective factor, thereby providing direct evidence for the optimization of combination treatment regimens. Lee et al. used whole-liver CT images without manual tumor annotation and integrated patient clinical characteristics and treatment information to construct a DL model based on pretreatment whole-liver CT images and clinical data. This model is used to predict the OS of patients with HCC after any initial treatment. It has achieved universal survival prediction across multiple treatment modalities^103^ and maintained good performance in an external CT cohort (C-index, 0.750).

### 3.5 Application of AI in clinical treatment decision-making

To accommodate the diversity of clinical treatments for HCC, the effectiveness of AI in assisting individualized decision-making is gradually being validated. Some researchers focus on developing models that can directly recommend preferred treatment options. For example, the ATOM framework proposed by Lin et al. has achieved significantly better predictions of efficacy and survival compared with traditional methods in external validation. When the model’s recommendations align with clinical decisions, patients achieve better outcomes.^104^ Similarly, the model constructed by Han et al. can classify patients with advanced HCC into different risk levels and provide corresponding treatment recommendations for each level.^105^ For treatment choices with competing options (such as TACE vs. HAIC), AI models assist decision-making by predicting differences in treatment efficacy. The DL model developed by An et al. specifically guides the choice between TACE and HAIC for patients with unresectable HCC and demonstrates good discriminative ability in predicting disease progression.^106^ In terms of surgical procedure selection for early-stage HCC (surgical resection vs. RFA), Shan et al. demonstrated the significant value of peritumoral radiomics features in predicting ER and weighing the two strategies.^107^ Liu et al. used a DL model based on ultrasound dynamic videos for “virtual secondary stratification” and identified patient subgroups more likely to benefit from another treatment approach.^108^ Another type of model does not directly recommend a treatment plan but provides core evidence for clinical selection by accurately predicting decisive prognostic events such as vascular invasion and recurrence. For instance, the model developed by Fu et al. can accurately predict the risk of major vascular invasion and its performance is not affected by factors such as the patient’s initial treatment plan.^109^ A more common research strategy is to directly predict the prognostic outcomes of different treatment options. The model developed by Choi et al. indirectly supports initial treatment selection by predicting the 5-year survival rates of different therapies.^110^ Other researchers have also constructed predictive models centered on the recurrence risk of different treatments.^111^

Across these modalities, distinct AI focuses emerge: TACE models often prioritize survival and response prediction using both static and longitudinal data, ablation models emphasize ER and post-procedural image interpretation, and TARE models explore dosiomics and normal-tissue radiomics. Despite modality-specific nuances, common challenges persist: data heterogeneity, lack of standardization, and limited generalizability. Simple radiomics models are more clinically ready for specific tasks (*e.g.*, predicting PTLF or ER after ablation), but complex multi-omics and longitudinal DL models, though less mature, offer deeper biological insights and potential for personalized sequencing. Bridging this gap requires hybrid approaches and rigorous multi-center validation.

## 4 Interpretability and future development directions of radiomics and DL

AI is expected to become a powerful tool for improving patient outcomes. However, behind the vigorous development of academic research, clinical translation lags far behind. Most models have not been validated in clear clinical or performance endpoints. A notable translational gap exists between high-performance research algorithms and reliable and useable clinical tools.

### 4.1 Clinical value-oriented verification and integration

The clinical utility of AI in HCC interventional therapy is currently limited by several factors. First, most models are developed using retrospective, single-center data from specific populations (*e.g.*, HBV-related HCC), leading to risk of overfitting and poor generalizability across different etiologies or ethnicities. Second, specialized datasets for interventional cohorts are significantly smaller than those for general HCC screening, hindering robust model development. Furthermore, the lack of standardization in image acquisition, segmentation, and evaluation criteria prevents objective performance comparisons and the establishment of consensus best practices.

To address these challenges, there is an urgent need for large-scale, multicenter, prospective databases. Implementing technical frameworks such as federated learning can facilitate cross-institutional data sharing while ensuring privacy and security. Future research must transcend single data dimensions by integrating radiomics and deep learning with multi-omics data (*e.g.*, genomics, transcriptomics, and pathology) to enhance the biological basis of personalized treatment strategies. Finally, adhering to standardized reporting guidelines, such as TRIPOD-AI and radiomics quality scores, is essential to ensure model transparency and clinical adoption. 112, 113, 114

### 4.2 Improvement and innovation of methodology

AI models, particularly DL models, lack transparency in their decision-making processes. Although post hoc interpretation methods, such as Shapley additive explanations (SHAP) values and class activation maps, can provide some insights,^67^^,^^68^^,^^76^^,^^38^^,^^40^^,^^115^ they often lack clear biological or clinicopathological associations, and cannot explain the fundamental connection between imaging features and clinical outcomes. This makes it difficult for doctors and patients to understand and trust a prediction result that cannot provide a reasonable explanation, which severely hinders the integration of models into clinical decision-making processes. In the future, more technical support will be needed to enhance the interpretability of algorithms. It should be noted that other types of non-visual explainable AI (XAI) methods also have great potential to provide diverse and in-depth insights, which are crucial for understanding and trusting AI systems. Holzinger et al. classified non-visual XAI methods into three categories based on their output forms: case-based explanations (such as counterfactual explanations), textual explanations (such as automatically generated diagnostic reports), and auxiliary explanations (such as quantifying prediction uncertainty). They emphasized that the focus of future research lies in effectively integrating these diversified XAI methods into clinical workflows and empirically evaluating their actual influence on medical decision-making.^116^

### 4.3 Construction of an ethical and regulatory framework

In the medical field, AI algorithms analyze vast amounts of personal data, which involves both personal privacy and public safety. It is imperative to interpret sensitive data without compromising confidentiality and privacy. Industry leaders, healthcare service providers, and regulatory agencies play crucial roles in formulating and adhering to responsible AI development standards. They collectively bear the responsibility of enhancing patient care quality without compromising patient safety, and must establish patient trust by convincingly demonstrating their commitment to safety, security, fairness, and privacy.^117^^,^^118^ Seyyed-Kalantari et al. found that AI-based systems exhibit systematic biases by consistently and selectively underestimating diseases among underserved populations in healthcare. These patients have a higher risk of missed diagnoses, which exacerbates existing health inequalities.^119^ Anderson proposed the establishment of independent quality assurance labs, drawing on third-party verification models such as Consumer Reports and UL Solutions, to externally verify the safety and effectiveness of AI tools. This initiative, based on a consensus-driven framework and using diverse and representative datasets for testing, is expected to ensure the fairness and clinical quality of AI models across different populations.^118^

## 5 Conclusion

AI, particularly imaging technologies integrating radiomics and DL, has demonstrated significant potential in efficacy prediction and precision management of HCC. However, successful clinical translation depends on more than increasing algorithmic complexity. The immediate priority is to systematically address the challenges of model interpretability, generalizability, and prospective clinical validation through interdisciplinary and interinstitutional collaboration.

Future efforts must focus on building a robust ecosystem encompassing standardized data sharing, innovative interpretable technologies, rigorous clinical evaluation, and responsible ethical frameworks. By bridging these gaps, AI can evolve from a research prototype into a reliable clinical assistant, ultimately achieving the fundamental goal of improving patient outcomes.

## CRediT authorship contribution statement

**Ying Lei:** Writing – review & editing, Writing – original draft, Conceptualization. **Tianyi Xia:** Writing – review & editing, Conceptualization. **Yawen Wang:** Writing – review & editing. **Xinyu Zhou:** Writing – review & editing. **Xinyu Gao:** Writing – review & editing. **Shenghong Ju:** Writing – review & editing, Supervision, Project administration.

## Informed consent

Not Applicable.

## Ethics statement

Not Applicable.

## Organ donation

Not applicable.

## Animal treatment

Not applicable.

## Data availability statement

Data sharing is not applicable to this article, as no new data were created or analyzed in this study.

## Declaration of generative AI and AI-assisted technologies in the writing process

During the preparation of this work, the authors used Nano Banana in order to assist with the layout and typesetting of Fig. 2. After using this tool, the authors reviewed and edited the content as needed and take full responsibility for the content of the published article.

## Funding

This work was supported by the National Natural Science Foundation of China (Grant 92359304, 82330060, 82427803 to Shenghong Ju, and Grant 823B2040 to Tianyi Xia), and the Research Personnel Cultivation Programme of Zhongda Hospital Southeast University (Grant CZXM-GSP-RC179 to Tianyi Xia).

## Declaration of competing interest

The authors declare that they have no known competing financial interests or personal relationships that could have appeared to influence the work reported in this paper.
